# Evaluation of prokaryotic diversity of five hot springs in Eritrea

**DOI:** 10.1186/s12866-017-1113-4

**Published:** 2017-09-22

**Authors:** Amanuel M. Ghilamicael, Nancy L. M. Budambula, Sylvester E. Anami, Tadesse Mehari, Hamadi I. Boga

**Affiliations:** 1 Institute for Biotechnology Research, Jomo Kenyatta University of Agriculture and Technology, P.O. Box 62000-00200, Nairobi, Kenya; 2 Embu University College, P. O. Box 6-60100, Embu, Kenya; 3 National Commission for Higher Education in Eritrea, Asmara, Eritrea; 4 Taita Taveta University, P.O. Box 635-80300, Voi, Kenya

## Abstract

**Background:**

Total community rDNA was used to determine the diversity of bacteria and archaea from water, wet sediment and microbial mats samples of hot springs in the Eastern lowlands of Eritrea. The temperatures of the springs range from 49.5 °C to 100 °C while pH levels varied from 6.97 to 7.54. Akwar and Maiwooi have high carbonate levels. The springs near the seashore, Garbanabra and Gelti, are more saline with higher levels of sodium and chlorides. Elegedi, situated in the Alid volcanic area, has the highest temperature, iron and sulfate concentrations.

**Results:**

The five hot springs shared 901 of 4371 OTUs recovered while the three sample types (water, wet sediment and microbial mats) also shared 1429 OTUs. The Chao1 OTU estimate in water sample was significantly higher than the wet sediment and microbial mat samples. As indicated by NMDS, the community samples at genus level showed location specific clustering. Certain genera correlated with temperature, sodium, carbonate, iron, sulfate and ammonium levels in water. The abundant phyla included Proteobacteria (6.2–82.3%), Firmicutes (1.6–63.5%), Deinococcus-Thermus (0.0–19.2%), Planctomycetes (0.0–11.8%), Aquificae (0.0–9.9%), Chlorobi (0.0–22.3%) and Bacteroidetes (2.7–8.4%).

**Conclusion:**

There were significant differences in microbial community structure within the five locations and sample types at OTU level. The occurence of Aquificae, Deinococcus-Thermus, some Cyanobacteria and Crenarchaeota were highly dependent on temperature. The Halobacterium, unclassified Thaumarchaeota, Actinobacteria and Cyanobacteria showed significant correlation with salinity occurring abundantly in Garbanabra and Gelti. Firmicutes and unclassified Rhodocylaceae were higher in the microbial mat samples, while Archaea were prominent in the wet sediment samples.

**Electronic supplementary material:**

The online version of this article (10.1186/s12866-017-1113-4) contains supplementary material, which is available to authorized users.

## Background

Thermal springs are scattered in the eastern low lands of Eritrea. The geochemical survey conducted by the Ministry of Energy and Mines in collaboration with the United States Geological Survey (USGS) has shown that a volcanic area called Alid, which is located 120 km South of Massawa, Eritrea’s main port city, is inundated with fumaroles and thermal pools [1]. The geothermal reservoir at Alid is vapour-dominated and surface thermal waters are derived from shallow, steam heated ground water reservoirs [2]. Geological, geochemical and hydrothermal studies have documented the existence of other thermal springs in the Eastern lowlands. They are Gahtelai, along the Asmara-Massawa highway as well as Garbanabra and Gelti near the Gulf of Zula [3]. The thermal springs near the Gulf of Zula are adjacent to the shoreline with water chemistry indicative of a large measure of mixing with seawater [3].

Despite numerous studies of bacterial communities in different natural habitats, exploitable microbial diversity is not exhaustive and microorganisms represent the largest reservoir of undescribed biodiversity [4]. Extreme environments harbour untapped microbial diversity with potential use as products and resources for biotechnological processes. Thermophilic bacteria have been isolated from hot springs, solfataric fields (a volcanic creator containing an opening through which hot sulphurous gases emerge), and hydrothermal vents throughout the world [5].

Amplicon sequencing, in particular that of the small subunit rRNA gene (16S rRNA gene in Bacteria and Archaea or 18S rRNA gene in Eukarya), is a widely applied approach to study the composition, organization and spatiotemporal patterns of microbial communities, due to its ubiquity across all domains of life [6]. In the last decades, 16S rRNA gene amplicons were analyzed using fingerprinting techniques such as TRFLP [7] and ARISA [8] in combination with clone library construction and Sanger sequencing. However, this method often provided insufficient coverage to describe and compare microbial communities [9]. Now, high-throughput sequencing (HTS) technology and the application of barcode indexing are allowing the collection of thousands of sequences from a large number of samples simultaneously [10, 11]. These approaches have revealed deeper insights into the diversity of microbial communities [12, 13] and, by increasing sample numbers, have expanded the possibilities to study community and population dynamics over much finer temporal [14] and spatial scales [12]. Our understanding of ecosystem functioning can be illuminated to a greater extent through an in-depth analysis of prokaryotic communities. Next generation sequencing (NGS) of hypervariable regions of the 16S rRNA gene, is a cost-effective and a better alternative to examine the phylogenetic diversity of microbial populations in different ecosystems.

Recently, microbial communities in thermal springs have been studied in different continents, such as those in, India [15–17], El Tatio Geyser Field, Chile [18], Yunnan and Tibet, China [19], Sungai Klah and Ulu Slim, Malaysia [20], Philippines [21] and Romania [22].

Different thermophilic environments have different microbial phenotypes due to different physical and chemical conditions, biogeography and geological history [23]. Even though Eritrea is endowed with many thermal hot springs, apart from their prospects in geothermal energy, the thermal hot springs in Eritrea have not yet been studied with respect to microbial diversity and biotechnological prospects. This is the first culture independent study of the microbial community within the hot springs from Eritrea. This study involved the use of Illumina sequencing of PCR products of 16S rRNA genes to obtain a less biased estimation of the microbial community in the hot springs. The study investigated the overall prokaryotic diversity present in water, wet sediment and microbial mat of five hot springs in Eritrea using NGS.

## Methods

### Study site

Samples analyzed in this study were collected from five hot springs: two hot springs; Maiwooi (15° 32′ 53″N 39° 06′ 38″ E) and Akwar (15° 33′ 34″N 39° 05′ 37″ E) located near Gahtelai at elevations of 330.1 and 344.5 m, temperatures of 51.9 and 49.0 °C and pH range of 7.54 and 6.97 respectively; two hot springs; Garbanabra (15° 03′ 38″N 39° 46′ 27″ E) and Gelti (15° 03′ 39″N 39° 46′ 46″ E) near Irafayle in the shore of Gulf of Zula at elevations of 0.0 and 0.0 m, temperatures of 51.0 and 52.7 °C and pH range of 7.05 and 7.01 respectively; and one hot spring; Elegedi (14° 52′ 55″N 39° 55′ 37″ E) located in Alid volcanic center at elevation of 512.7 m, temperature of 100 °C and pH range of 7.19. From each hot spring, triplicate samples of water, wet sediment and microbial mat were collected.

Sampling was conducted three times in a day, that is, morning from 7:00 A.M to 9:00 A.M, noon from 12:00 P.M to 1:00 P.M and after noon from 4:00 P.M to 6:00 P.M. From each hot spring, water samples were collected using autoclaved 500 ml glass reagent bottles with screw top lids. Approximately 500 g of wet sediment samples were collected from the upper sediment and placed in 500 ml autoclaved bottles by scooping using sterile spoon. Mat samples were aseptically scraped using a sterile spatula into sterile zip lock plastic bags. For chemical analysis, water samples of 1000 ml as well as 500 g of wet sediment samples were collected from each hot springs. All samples were preserved on dry ice immediately after sampling, and transported to the laboratory in Quality Control Laboratory (QCL), Massawa, Eritrea.

### Chemical analysis of the water and wet sediment samples

The water and wet sediment samples collected from each hot spring for chemical analysis and were stored at 4 °C until chemical analysis was done. Analyses of phosphate, potassium, calcium, magnesium, sulfate, ammonia, sulfur, sodium, iron, manganese, copper, boron and zinc were conducted at the Crop Nutrition Laboratory Services Ltd. (Cropnuts, Nairobi, Kenya).

### DNA extraction from water, sediment and microbial mat samples

The water, wet sediment and algal mat samples collected from each hot spring were stored at −80 °C until DNA extraction was done. Community DNA was extracted from water, sediment and microbial mat samples as described by Kambura et al. [24]. From the wet sediment and microbial mat samples, 0.5 g from each of the wet sediment and microbial mat samples, were placed in 1.5 ml Eppendorf tubes. Approximately 500 ml of the water samples was filtered through 0.22 μm Whatman filter papers and centrifuged. All water samples formed a visible pellet upon centrifugation. The pellet, from each of the water samples, was re-suspended in 5 ml of phosphate-buffered saline solution. After three successive washings in phosphate-buffered saline solution, the pellet was placed in 1.5 ml Eppendorf tubes. DNA was extracted from each sample using the phenol chloroform method [25]. Extracted DNA pellets were air dried and stored at −20 °C.

### Amplicon library preparation

PCR amplification of the 16S rRNA gene V4-V7 variable region was carried out from extracted DNA using primers 515F (GTGCCAGCMGCCGCGGTAA) that had barcode and 806R (GGACTACHVGGGTWTCTAAT) according to Caparaso et al. [26]. PCR amplification was carried out in 30 cycles using the HotStarTaq Plus Master Mix Kit (Qiagen, USA) under the following conditions: 94 °C for 3 min of initial heating, followed by 30 cycles of 94 °C for 30 s, 53 °C for 40 s and 72 °C for 1 min, after which a final elongation step at 72 °C for 5 min was performed. The quality of PCR products was assessed on 2% agarose gel to determine the success of amplification and the relative intensity of bands. Multiple samples, tagged with different barcodes, were pooled together in equimolar ratios based on their DNA concentrations from the gel images. Pooled samples were purified using calibrated Ampure XP beads (Beckman Coulter) for use in library preparation. The pooled and purified PCR product was used to prepare the DNA library by following Illumina TruSeq DNA library preparation protocol [27]. Sequencing was performed at Molecular Research DNA (www.mrdnalab.com, Shallowater, TX, USA) on a MiSeq 2x300bp Version 3 following the manufacturer’s guidelines.

### Sequence analysis, taxonomic classification and data submission

Sequences obtained from the Illumina sequencing platform were depleted of barcodes and primers using a proprietary pipeline (Molecular Research DNA, Shallowater, Texas) developed at the service provider’s laboratory. Low quality sequences were identified by denoising and filtered out of the dataset [28]. Short sequences <200 base pairs after phred20- based quality trimming, sequences with ambiguous base calls, and those with homopolymer runs exceeding 6 bp were removed. Sequences were analyzed by a script optimized for high-throughput data to identify potential chimeras in the sequence files, and all definite chimeras were depleted as described previously [29]. Operational taxonomic units (OTUs) were defined by clustering at 3% divergence (97% similarity). All this data filtering was done by the service provider using their pipeline. Taxonomy was assigned to each OTU using BLASTn against a curated database derived from GreenGenes, RDPII, SILVA SSU Release 119 and NCBI [30]. Resulting raw sequences were submitted to NCBI Sequence Read Archive with study accession number SRP064297.

### Statistical analysis

Diversity indices (Richness, Shannon, inverted Simpson, absolute diversity), rarefaction curves and Venn diagram (to compare the shared OTUs between the hot springs and water samples) were calculated from the resulting OTUs using Vegan package version 1.16–32 in R software version 3.1.3 [31]. Hundred iterations of rarefaction was computed for each sample to 20,000 sequence using QIME pipeline Version 1.8.0 [32]. Chao1, a non-parametric estimation of OTU richness between the data sets calculated from the rarified data was used to compare species richness between the data sets, hot springs and sample types. Kruskal-Wallis rank sum test and pairwise Wilcox test were used to compare between the datasets, hot springs and sample types from the Chao1 OTU estimation using R programming language [31]. Community and Environmental distances were compared using Analysis of similarity (ANOSIM) test, based upon Bray-Curtis distance measurements with 999 permutations. Significance was tested at 95% confidence interval (*p* = 0.05).

Non-metric Multidimensional Scaling (NMDS), redundancy analysis (RDA) as well as Hierarchical clustering of the environmental data, based on Euclidian, and the community data, based on Bray-Curtis dissimilarity, were carried out using the R programming language [31] and the Vegan package [33]. Correlation, based on Pearson’s correlation coefficient, analysis between the environmental samples and the community structure was conducted and significance was tested using Mantel test in R programming language [31]. To support OTU-based analysis, taxonomic groups were derived from the number of reads assigned to each taxon at all ranks from domain to species using the taxa_summary.txt output from QIIME pipeline Version 1.8.0.

## Results

### Characteristics of the investigated hot springs

The five hot springs were selected based on distinct characteristics such as temperature, salinity and location. The physicochemical parameters of each of the hot springs are presented in Additional file 1: Table S1.

Hierarchical clustering of the physiochemical attributes of the water samples based on Euclidian distance matrix showed three distinct clusters according to temperature and geographical location (Additional file 2: Figure. S1A). Akwar and Maiwooi, situated close to each other, have clustered together while Garbanabra and Gelti, which were characterized by higher salinity levels, due to influx of seawater from the nearby seashore, were grouped together. Elegedi, characterized by high temperature (100 °C), clustered separately from the other groups. High ammonium (190 mg/L) and sulfate (949 mg/L) levels were also recorded from Elegedi.

Hierarchical clustering of the wet sediment chemical parameters revealed two principal clusters (Additional file 2: Figure S1B). Garbanabra and Gelti, which are located on the seashore, were distinct from the others, that is, Maiwooi, Akwar and Elegedi. Surprisingly, according to sediment chemistry, Akwar was observed to be closely related to Elegedi than Maiwooi. Higher levels of sodium and calcium were recorded in Garbanabra (1160 and 143 ppm, respectively) and Gelti (826 and 1230 ppm, respectively) (Additional file 1: Table S1). Elegedi has registered greater levels of Sulphur (728 ppm), phosphorus (4.28 ppm), iron (404 ppm), copper (5.28 ppm), zinc (44.4 ppm) and manganese (101 ppm) levels.

### Assemblage and diversity of the prokaryotic communities

A total of 890,752 sequence reads greater than 200 bp were attained, after removing chimeras from 14 DNA amplicon data sets. Total OTU richness (3% distance) was observed to be 4371. OTUs per data set ranged between 376 and 2354 (Additional file 3: Table S2). As indicated by rarefaction analysis (Additional file 4: Figure S2), sequencing depth was far from being exhaustive even in the largest dataset.

**Fig. 1:**
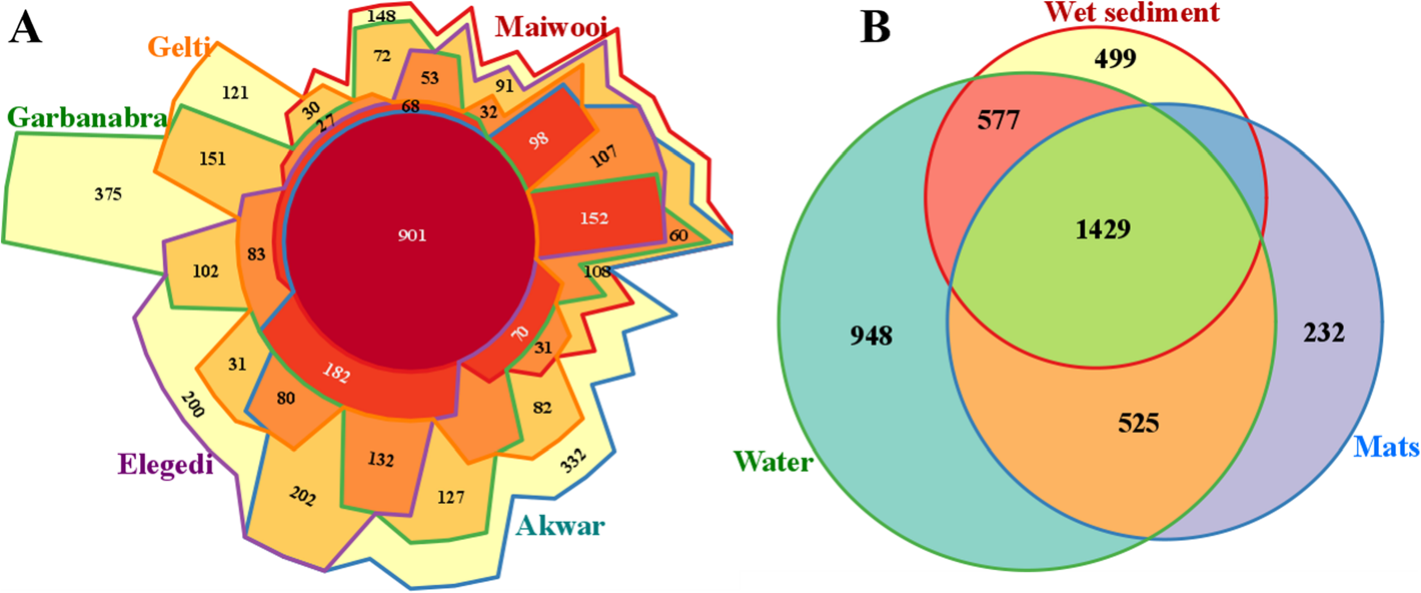
The distribution of shared OTUs across the five hot springs in Eritrea and the three Sample types. **a** and **b** Venn diagrams showing the distribution of unique and shared OTUs within various sample types in the five hot springs

The distribution of shared OTUs across the hot springs and the sample types is shown in Fig. 1. Larger proportions of OTUs were shared between the five hot springs (901) as well as the three sample types (1429). The distribution of shared OTUs across the sample types revealed greater overlap between water and wet sediment samples (Fig. 1b). There was relatively lower overlap between the microbial mat and wet sediment sample types. Statistical analysis based on ANOSIM revealed significant differences in OTUs composition between the three locations (*r* = 0.28, *p* = 0.011) as well as the sample type (*r* = 0.53, *p* = 0.001).

### Microbial richness

**Fig. 2:**
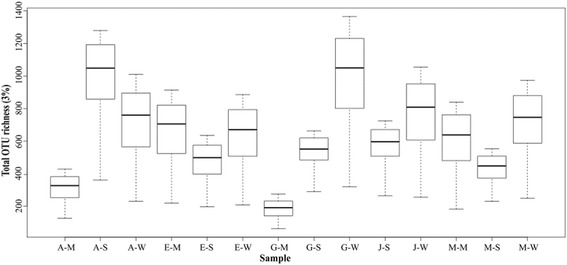
Chao1 estimates of OTU richness in the datasets using rarefied data. The first letters of the sample names refer to the five hot springs (A = Akwar, E = Elegedi, G = Garbanabra, J = Gelti and M = Maiwooi), while the second letters are for sample types (A = Microbial mat, S = wet sediment, and W = water)

Chao1, a non-parametric estimation of OTU richness as calculated from the alpha-rarefaction data, was used to compare species richness between the datasets (Fig. 2).

Significant difference (Kruskal-Wallis chi-squared = 339.6, *p* < 2 × 10^−7^) of the Chao1 estimates was observed between the datasets. However, pairwise comparisons using Wilcoxon rank sum test of the Chao1 OTU estimates revealed the difference between some of the datasets was not significant.

### Abundance of prokaryotic taxa

**Fig. 3:**
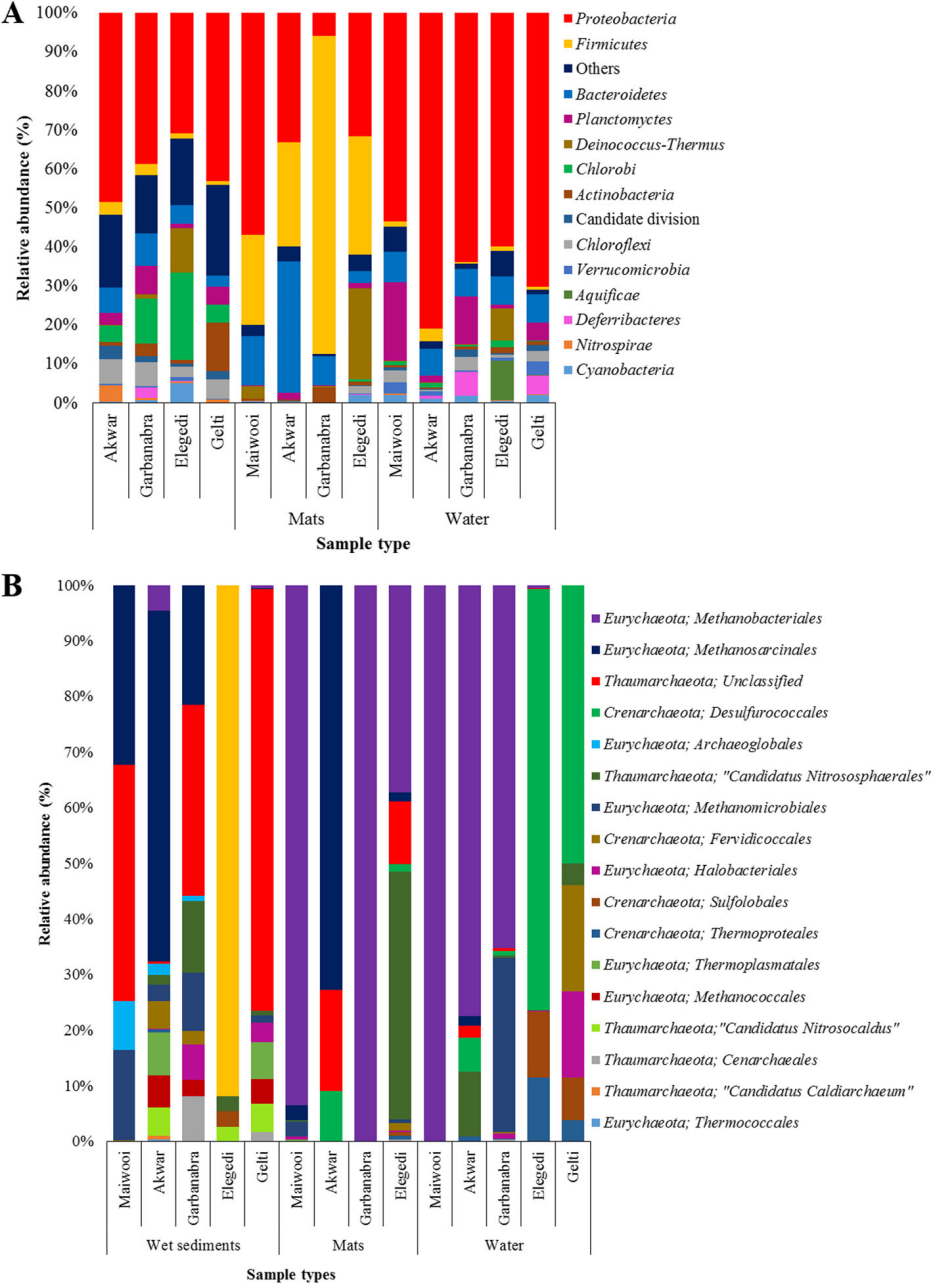
Relative abundance of bacteria at phyla level **a** and archaea at order level in various samples collected from the hot springs in Eritrea **b**

The majority of the sequences (95.1%) were observed to belong to the kingdom bacteria. These results suggest that bacteria are the most dominant taxa in all five hot springs. There were 49 phyla affiliated with bacteria in the present study (Fig. 3a). The outcomes of the present study revealed that the bacterial communities were highly diverse (Additional file 5: Table S3) and the prominent groups were *Proteobacteria* (6.2–82.3%), *Firmicutes* (1.6–63.5%), *Bacteroidetes* (2.7–8.4%), *Deinococcus_Thermus* (0.0–19.2%), *Planctomycetes* (0.0–11.8%), *Chlorobi* (0.0–22.3%), *Chloroflexi* (0.0–2.2%), *Verrucomicrobia* (0.0–3.4%) and *Euryarchaeota* (0.0–7.7%).

Under the bacterial kingdom, OTUs were distributed in 272 families. Among the most abundant were *Pseudomonadaceae* (8.7%), *Comamonadaceae* (5.2%), *Thermaceae* (5.1%), *Caulobacteraceae* (4.9%), *Sphingomonadaceae* (4.3%), *Bacillaceae* (4.1%), *Rhodospirillaceae* (3.4%), *Planctomycetaceae* (3.3%) and *Rhodobacteraceae* (2.7%) as shown in Additional file 6: Table S4.

The phylum *Proteobacteria* was the most abundant phylum observed in all the five hot springs. It was represented by five classes and 95 families. Statistical analysis based on ANOSIM, revealed significant difference in the composition of this phylum between the sample types (*r* = 0.341, *p* = 0.008). Higher abundance of *Proteobacteria* was observed in the water samples. The relative abundance of *α-Proteobac*teria and *γ-Proteobacteria* from the water samples was higher in the saline hot springs, Garbanabra (31.4% and 29.4%, respectively) and Gelti (38.7% and 19.5%, respectively) compared to *β-Proteobac*teria.

The phylum *Firmicutes* was the second most abundant phylum observed. The relative abundance of this phylum ranged from 1.5% (in water sample from Gelti) to 63.5% (in microbial mat sample from Garbanabra). Significantly higher numbers of *Firmicutes* were detected from the microbial mat samples (*r* = 0.583, *p* = 0.003). They were abundant in the microbial mat samples (32.3%) compared to wet sediment and water samples (5.9% and 2.4%, respectively).

*Deinococcus–Thermus*, a phylum of bacteria that are highly resistant to environmental hazards, were detected at fairly low relative abundance (0.0–7.2%). The sequences retrieved affiliated to this phylum were from the two orders; *Thermales* (5.1%) and *Deinococcales* (0.5%). The order *Thermales* was detected more abundantly from the high temperature hot spring (100 °C), Elegedi and the specific composition of this group has showed significant correlation with temperature (*r* = 0.468, *p* < 0.05). Nine genera from phylum *Planctomycetes* were retrieved, among which, *Planctomyces* was the dominant genus. The microbial composition of the group was significantly different between the sample types (*r* = 0.285, *p* = 0.002) and were abundantly present in the water samples. The actinobacterial communities in the Eritrean hot springs were diverse (0.6–4.5%). According to ANOSIM, the difference in similarity of actinobacterial composition between the sample types was significant (*r* = 0.178, *p* = 0.032).

The filamentous anoxygenic photosynthetic phylum *Chloroflexi* in Eritrean hot springs was dominated by the class *Caldilinea*. *Chloroflexi* were observed inhabiting in the five hot springs with different temperature ranges. Significance was observed in the distribution of *Chloroflexi* between the sample types (*r* = 0.424, *p* = 0.003). This group of bacteria was encountered in greater abundance in the water and wet sediment samples compared to microbial mat samples. The phylum *Nitrospirae* was recovered from the hot springs in Eritrea at a very low relative abundance (0.0–4.6%). They were detected at relatively higher abundances from the wet sediment sample of Akwar.

Some Archaeal sequences were also detected (1.5%), which were affiliated with *Euryarchaeota* (62.3%), *Crenoachaeota* (25.8%) and *Thaumarchaeota* (12.2%). Based on ANOSIM, the archaeal community structure was significantly different between the three sample types (*r* = 0.236, *p* = 0.008). Archaeal community structure of the wet sediment samples was distinct from the other two sample types. The dominant archeal phylum *Euryarchaeota* (Additional file 7: Table S5) included *Methanosarcinales* (32.7%), *Methanobacteriales* (15.2%) and *Methanomicrobiales* (4.1%). Included in phylum *Crenarchaeota* were *Desulforococcales* (12.4%), *Thermoproteales* (2.1%) and *Archaeglobules* (1.5%) as shown in Fig. 3b. While the ammonia oxidizing archaea, *Thaumarcheota* included, among others, Uncultured *Thaumarchaeota* (2.1%), “*Candidatus* Nitrosocaldus” (1.8.0%), and “*Candidatus* Nitrososphaera” (1.6%).

Twenty one candidate divisions were retrieved from all the hot springs. The candidate division nbk19, particularly, had shown significant correlation with salinity (*r* = 0.248, *p* = 0.021). There was significance, based on ANOSIM, in the Candidate division composition between the sample types (*r* = 0.372, *p* = 0.001). Of the 16S rRNA filtered reads recovered from the wet sediment samples, 4.5% could not be classified into any phylum under the Bacteria domain while only 1.2% from the water samples could not be classified (Additional file 5: Table S3).

### Microbial community composition

**Fig. 4:**
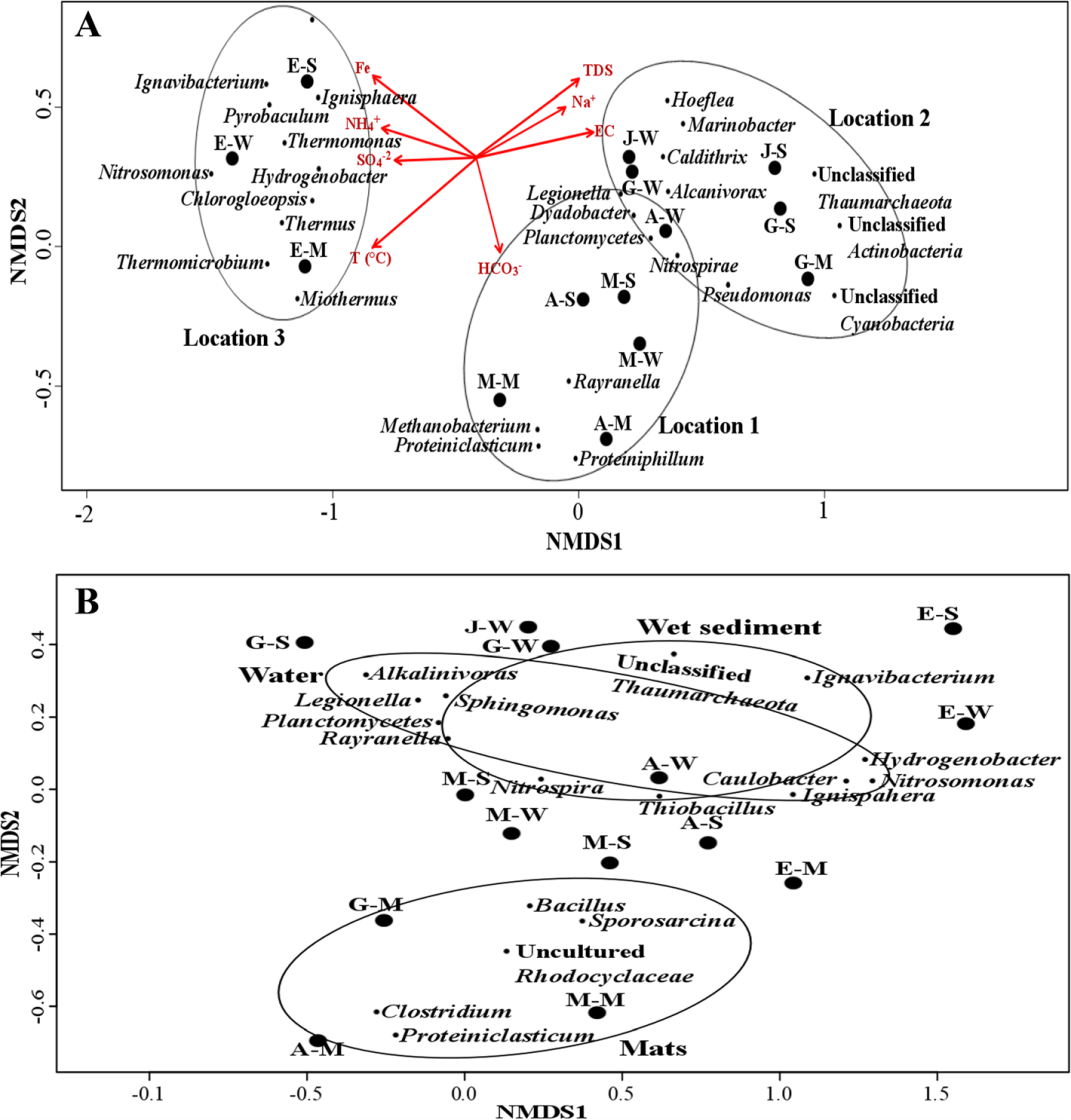
NMDS based on Bray-Curtis dissimilarities between microbial compositions, at genus level, grouped according to the locations **a** and sample types **b**. A bi-plot is overlaid on the ordination to the displayed geochemical variables that are correlated with the microbial community structure. Only variables that have a significant correlation (*P* < 0.05) are shown. The abbreviations represent the datasets. First letters refer to the five hot springs (A = Akwar, E = Elegedi, G = Garbanabra, J = Gelti and M = Maiwooi), while the second letters are for sample types (A = mats, S = wet sediment, and W = water)

As indicated by NMDS, the community composition at genus level clustered according to the locations of the hot springs (Fig. 4a). Location 1 (Akwar and Maiwooi), Location 2 (Garbanabra and Gelti), and Location 3 (Elegedi).

In Location 1 (Akwar and Maiwooi), the archaeal community was mainly composed of sequence reads closely related to euryarchaeal genus *Methanomicrobium* as well as bacterial genera to *Proteiniphilium* from the phylum *Bacteroidetes* and *Proteiniclasticum* from *Firmicutes*. The distribution of these genera positively correlated with bicarbonate levels (*r* = 0.62, 0.32 and 0.26 respectively at *p* < 0.05). The genus *Proteiniclasticum*, a strictly anaerobic proteolytic bacteria, was the most abundant. It was detected in greater numbers from the microbial mat samples of Akwar and Maiwooi (7.6 and 10.9%).

Location 2 (Garbanabra and Gelti) were mainly associated with unclassified *Thaumarchaeota*, *Actinobacteria* and *Cyanobacteria* indicating possible occurrence of novel microorganisms in these springs. The genera *Hoeflea* from *α-proteobacteria*, *Pseudomonas*, *Marinobacter* and *Alcanivorax* from *γ-proteobacteria* and *Caldithrix* from *Deferribacteres* were prominent in these two springs and their abundances positively correlated with marine geochemical indictors such as sodium and calcium. Sequences affiliated to the genus *Bacillus* were recovered at higher abundance from the microbial mat samples of Garbanabra (36.2%, respectively). The relative abundance of *Bacillus* in the other samples ranged from 0.1 to 3.0%.

The boiling hot spring in Location 3, characterized by having an extremely high temperature (100 °C) displayed a diverse thermophilic population. Phylum *Aquificae* showed significant correlation with temperature (*r* = 0.36, *p* = 0.037). The highest relative abundance (9.9%) was observed from the water sample of the boiling hot spring, Elegedi. Two genera of Aquificae, *Hydrogenobacter* and *Thermocrinis* were detected. *Hydrogenobacter* was the most dominant genus. *Thermocrinis* were recovered at much lower abundance than *Hydrogenobacter* from Elegedi. *Meiothermus*, the dominant genera from *Deinococcus-Thermus*, and *Thermus* were also detected in relatively high abundances from the boiling hot spring. *Meiothermus* was the dominant genus (19.5%) in the mat samples of Elegedi. The relative abundance of the phylum *Chlorobi* (0.0–9.9%) was dominated by the chemoheterotrophic genus, *Ignavibacterium*. *Ignavibacterium* was detected prominently from the wet sediment sample of Elegedi and its abundance correlated significantly with temperature (*r* = 0.422, *p* < 0.02). Sequences showing 93% similarity to the genus *Chlorogloeopsis* were also recovered predominantly from the mat (2580) and wet sediment (1072) samples of Elegedi. This cyanobacterial genus has shown positive correlation with temperature (*p* = 0.72, *p* = 0.002).

The archaeal community composition in Location 3 contained *Ignisphaera*, *Acidianus* and *Pyrobaculum.* The genus *Ignisphaera*, from the order *Desulfurococcales* was recovered at a relatively higher abundance (2.0%) from the water sample of the boiling hot spring. Its abundance was shown to correlate with temperature (*p* = 0.53, p = 0.002) and Sulphate levels. The abundance of *Desulfurococcales* (1701) was much higher than *Thermoproteales* (255) and *Sulfolobales* (269).

Sequences affiliated with *Thermomonas*, member of the *γ-proteobacteria*, as well as *Nitrosomonas*, from *β-proteobacteria*, were recovered from the water sample of Elegedi. The corresponding abundances of *Thermomonas* showed positive correlation with ferrous levels (*r* = 0.44, *p* < 0.012). The autotrophic ammonia-oxidizing bacteria *Nitrosomonas* also showed positive correlation with ammonia levels (*r* = 0.56, *p* = 0.011).

**Fig. 5:**
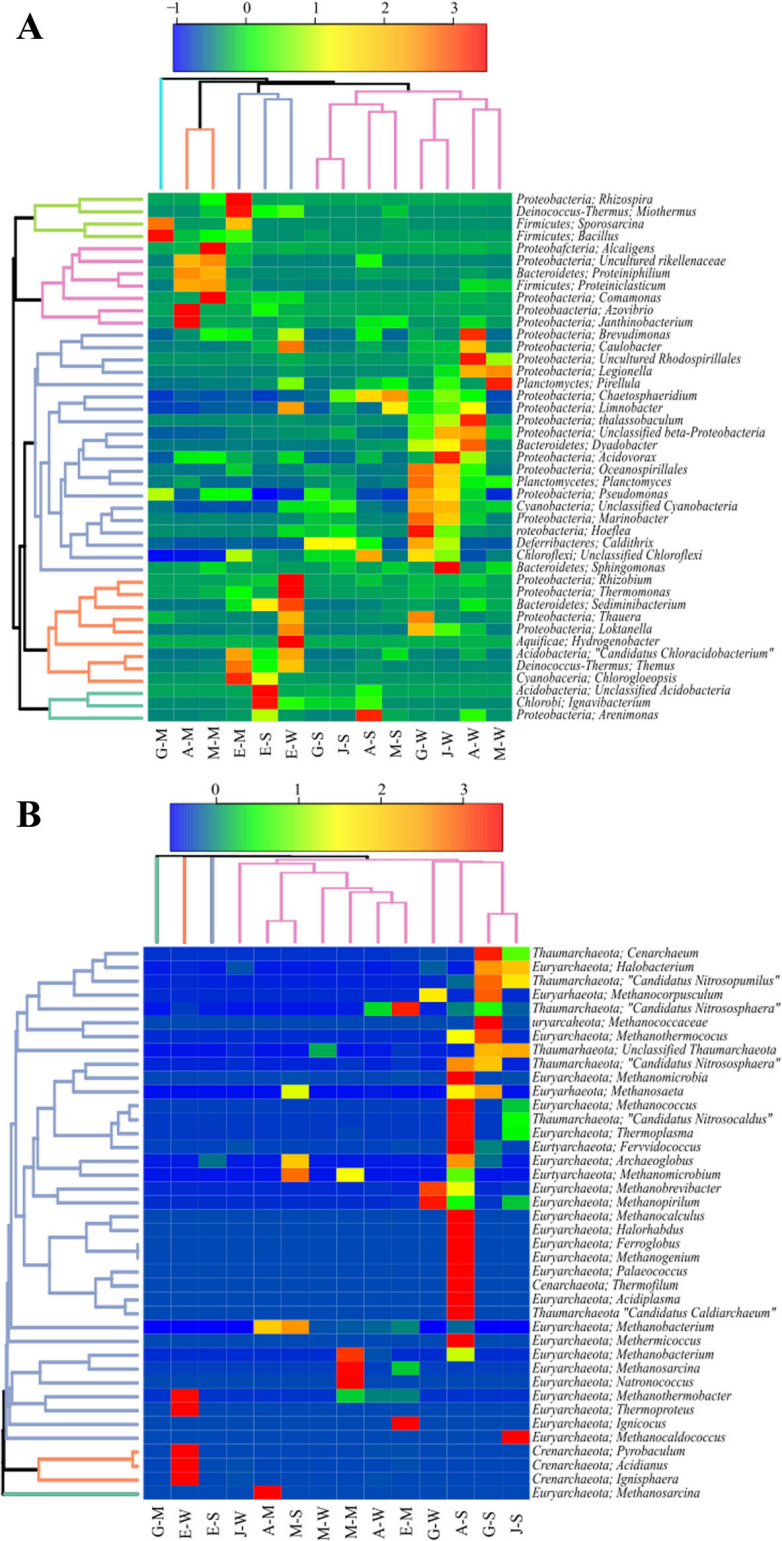
The hierarchical clustering based on Bray-Curtis dissimilarity, between the various samples, of the abundant taxa at genus rank for bacteria **a** and the abundant archaea **b**. The abbreviations represent the datasets. First letters refer to the five hot springs (A = Akwar, E = Elegedi, G = Garbanabra, J = Gelti and M = Maiwooi), while the second letters are for sample types (A = mats, S = wet sediment, and W = water)

The NMDS analysis of sample types showed an overlap in microbial communities between water and wet sediment samples (Fig. 4b). While, microbial mat samples formed a separate cluster. Hierarchical clustering, based on Bray-Curtis dissimilarity, of the abundant taxa at genus rank for bacteria and archaea in each of the three sample types is shown in Fig. 5. As it was evident in NMDS, the composition of the bacterial genera showed clustering based on locations of the hot springs (Fig. 5a). The heat map depicting the hierarchical clustering of archaeal genera showed a different pattern than bacterial genera (Fig. 5b).

## Discussion

The five hot springs investigated in the present study were highly diverse in their environmental attributes, encompassing a temperature range from 49.0 to 100 °C and salinity from 3 to 32 as well as wide ranges of water and sediment geochemistry. Maiwooi and Akwar have low salinity levels and are located at the main eastern escarpment in Gahtelai along the Asmara-Massawa highway with temperatures ranging from 50 °C to 70 °C. These low energy hot springs discharge near-neutral bicarbonate waters [2]. Gelti and Garbanabra are located on the seashore of the Red Sea near the Gulf of Zula. The salinity (3.2 and 3.1%, respectively) is much higher than Maiwooi and Akwar (0.4 and 0.7%, respectively) due to mixing with the sea water. Elegedi is located about 30 km away from the seashore of the Gulf of Zula and is associated with a high temperature geothermal system underlying the Alid volcanic centre in the Northern Danakil depression of Eritrea [2]. The bubbling water discharged from this hot spring is typical of the fumarolic steam condensate with high temperatures.

The majority of the sequences (95.1%) were observed to belong to the kingdom bacteria. These results suggest that bacteria are the most dominant taxa in all five hot springs. The phylotypes *Proteobacteria*, *Firmicutes*, *Deinococcus-Thermus*, *Planctomycetes*, *Bacteroidetes*, *Chloroflexi* and Aquificea have also been reported to inhabit hot springs in India [15–17], Yunnan and Tibet in China [34], Siloam in South Africa [35], and Malaysian hot spring [20]. The abundance of *Proteobacteria, Firmicutes, Bacteroidetes*, *Actinobacteria*, *Cyanobacteria* and *Chloroflexi* was also reported in the hypersaline hot springs of Lake Magadi and Little Magadi in Kenya [24].

The phylum *Proteobacteria* was the most abundant phylum observed in all the five hot springs. *Proteobacteria* has also been reported from many studies based on the 16S rRNA analysis of hot springs with moderately high and very high temperatures (44–110 °C) at various geographical locations [15, 17, 20, 21, 24, 36–38]. The relative abundance of *α-Proteobac*teria and *γ-Proteobacteria* from the water samples was higher in the saline hot springs compared to *β-Proteobac*teria. Previous investigation in the Baltic Sea revealed transitions in bacterial communities along the 2000 km salinity gradient [12]. The relative abundance of *α-Proteobac*teria and *γ-Proteobacteria* increased with salinity, whereas *Actinobacteria* and *β-Proteobac*teria displayed the opposite trend. The phylum *Firmicutes* was the second most abundant phylum observed. It was abundant in the microbial mat samples compared to wet sediment and water samples. This could imply that the *Firmicutes* sequences retrieved in the present study probably represent bacteria embedded in the microbial mat sample. Recently, high relative abundance of *Firmicutes* (23%) were observed from microbial mat samples at 45 °C [24].

Sequence affiliated with *Euryarchaeota*, *Crenoachaeota* and *Thaumarchaeota* were detected from the archaeal kingdom. The most abundant archaeal group *Euryarchaeota*, particularly the genus *Methermicoccus*, a thermophilic and methylotrophic methanogen [39], was predominantly recovered from the wet sediment sample in Akwar. Within the order *Thermoproteales*, the terrestrial genera *Thermofilum* was recovered only from the wet sediment sample of Akwar. *Thermofilum* was observed in YNP springs with mildly acidic pH (5.5–6.1) and moderate to high temperature, which is consistent with the physiology of this genus [40]. *Acidianus*, was the only genus observed from the order *Sulfolobales*. *Acidianus*, unlike the obligate aerobic *Sulfolobus*, is facultative anaerobic and can utilize sulfur either as an electron donor (in aerobic metabolism) or electron acceptor (in anaerobic metabolism). Garbanabra and Gelti, situated at the sea shore, where high levels of salinity was registered, harbored the genus *Halobacterium* (“salt” or “ocean bacterium”). This was confirmed by Mantel test, where the relative abundance of *Halobacterium* increased with increased salinity levels (*r* = 0.75, *p* = 0.02). The abundance of *Halobacterium* in these two hot springs was observed from the wet sediment samples.

At genus level, the microbial community composition clustered according to the three locations. In Location 1, euryarchaeal genus *Methanomicrobium* as well as bacterial genera *Proteiniphilium* from the phylum *Bacteroidetes* and *Proteiniclasticum* from *Firmicutes* were dominant. Location 2 were mainly associated with unclassified *Thaumarchaeota*, *Actinobacteria* and *Cyanobacteria* indicating possible occurrence of novel microorganisms in these springs. The locations of these two saline springs to the Gulf of Zula, suggest that a marine influence may be a possible reason for the occurrence of novel microorganisms in these springs. Similar observation was noted in hot springs in Philippines [21]. They observed large sequence reads in the two saline hot springs located close to Albay Gulf which were mainly associated with unclassified *Archaea* (3–19%), *Crenarchaeota* (63–82%), and *Bacteria* (25–58%), indicating possible occurrence of novel microorganisms in these springs.

The boiling hot spring in Location 3 displayed a diverse thermophilic population. Two genera of Aquificae, *Hydrogenobacter* and *Thermocrinis* were detected. *Hydrogenobacter* was the most dominant genus. Likewise, In Tengchong springs, *Hydrogenobacter* was the dominant genus from *Aquificae* in high temperature (73.8–93.6 °C) and circumneutral to alkaline pH (6.7–9.4) springs [41]. *Thermocrinis* were recovered at much lower abundance than *Hydrogenobacter* from Elegedi. The genus *Thermocrinis* was commonly found in other hot springs worldwide with near-neutral pH, high temperature (75–92 °C), and low sulfide concentrations (usually, 1 mg/L) [42–44]. The high sulphate concentration (949 mg/L) at Elegedi might have been the reason for the low numbers of *Thermocrinis*. *Meiothermus*, the dominant genera from *Deinococcus-Thermus*, and *Thermus* were also detected in relatively high abundances from the boiling hot spring. *Meiothermus* was the dominant genus (19.5%) in the mat samples of Elegedi. Genera *Thermus* and *Meiothermus* have been detected in hot springs with moderate-high temperatures of 50–99 °C and slightly acidic to alkaline pH, such as in Iceland [42], Kamchatka in Russia [45], Long Valley Caldera [46], the Great Basin of the United States [47], and Thailand [48]. However, *Thermus* and *Meiothermus* are usually not dominant in terrestrial hot springs except for the hot springs of Tibetan plateau [49] and a few springs in Iceland [42]. The predominance of *Meiothermus* over *Thermus* in the boiling hot spring is surprising, because most species of *Thermus* have been shown to grow anaerobically in the presence of nitrate with an optimal growth temperature of 65–75 °C, whereas *Meiothermus spp.* have a lower optimal growth temperature 50–65 °C and exhibit O_2_ respiration [50]. The genus *Chlorogloeopsis* were also recovered predominantly from the mat and wet sediment samples of the boiling hot spring. The upper temperature limit for *Chlorogloeopsis* was previously determined to be between 60 and 65 °C [51], while the water temperature of the boiling hot spring during sampling was 100 °C. Further investigation may be necessary to explain their occurrence.

The archaeal community composition in Location 3 contained *Ignisphaera*, *Acidianus* and *Pyrobaculum.* The abundance of *Desulfurococcales* was much higher than *Thermoproteales* and *Sulfolobales*. The predominance of *Desulfurococcales* in the boiling hot spring with high temperature (100 °C) and near-neutral pH (7.19) is consistent with the known hyperthermophily of these organisms [41]. Similar to *Desulfurococcales*, *Thermoproteales*, mainly *Pyrobaculum* relatives were the most abundant in Elegedi with high temperature and near-neutral pH. The high temperature of Elegedi hot spring is consistent with the hyperthermophilic nature of the genus *Pyrobaculum* (74–102 °C) with optimum 100 °C [52].

Surprisingly, sequences affiliated with *Thermomonas*, member of the *γ-proteobacteria*, as well as *Nitrosomonas*, from *β-proteobacteria*, were recovered from the water sample of Elegedi. Strains affiliated to the genus *Thermomonas* has been described previously as ferrous iron-oxidizing and nitrate-reducing bacteria [53]. *Thermomonas* has been described to grow between 37 and 50 °C [54] and moderately thermophilic *Nitrosomonas* at 50 °C [55] hence their detection in the boiling hot springs is surprising. Earlier, a culture of thermophilic nitrifying bacteria belonging to the genus *Nitrosomonas*, isolated from geothermal springs of Kamchatka (50–86 °C, pH 6.3–7.5) and Tajig (50–85 °C, pH 7.3–8.5), was shown to oxidize ammonium nitrogen at 40–70 °C [56]. Some previous studies have also reported other mesophilic bacterial species in high-temperature hot spring in the Philippines [21], where sequences affiliated to *Sulfobacillus* predominated in high-temperature spring BAL-0 (90.8 °C) and its occurrence was confirmed by the full-length bacterial 16S rRNA gene. The genus *Acinetobacter*, reported to have an optimum growth temperature between 33 and 35 °C [57], was recovered predominantly (92.1%) from extremely high temperature Tattapani hot spring (98 °C) in Central India [15]. Recently, *Alishewanella*, a genus from *γ-proteobacteria*, was also detected predominantly (4.1%) from a high temperature (95 °C) Soldhar hot spring in India using high throughput sequencing [37]. This might suggest the possible higher temperature tolerance of some bacteria known to be mesophiles. In the natural environment, biofilms enable bacterial consortium tolerate extreme temperatures and other environmental factors. Further investigations on the presence of high temperature tolerant *Nitrosomonas*, detected in boiling hot spring with high ammonium concentrations, are necessary to rule out dead or inactive cells carried in from the fringes of the boiling hot spring.

The NMDS analysis of sample types showed an overlap in microbial communities between water and wet sediment samples. While, microbial mat samples formed a separate cluster. Even though, there was notable overlapping between the wet sediment and water samples, the microbial community secured from water samples were commonly found elsewhere in the environment, whereas an appreciable part of the microbial community from the wet sediment samples remained unidentified and therefore might be environmentally unique. Of the 16S rRNA filtered reads recovered from the wet sediment samples, 4.5% could not be classified into any phylum under the Bacteria domain while only 1.2% from the water samples could not be classified. A similar observation was made in a study conducted in China which compared planktonic and sedimentary bacteria diversity of the Lake Sayram during summer [58]. In contrast to the planktonic bacteria, they detected an appreciable portion of the sedimentary bacteria that could not be classified into any known taxonomic unit.

## Conclusion

This study has documented, for the first time, the microbial community in five thermal springs from Eritrea using next generation sequencing. The five hot springs investigated in the present study were highly diverse in their environmental attributes, encompassing a temperature range from 49 to 100 °C and salinity from 0.3 to 3.2% as well as wide ranges of water and sediment geochemistry. The overall diversity and richness observed within the hot springs were higher than those reported before from other environments such as soil, deep-sea hydrothermal environments and acidic hot springs [41]. The overall microbial diversity at genus showed significance between the three locations of the hot springs NMDS analysis of sample types showed an overlap in microbial communities between water and wet sediment samples, while, microbial mat samples formed a separate cluster.

The majority of the sequences (95.1%) were observed to belong to the kingdom bacteria. These results suggest that bacteria are the most dominant taxa in all five hot springs. The bacterial phylum *Proteobacteria* were dominant in all samples. The specific compositions of *Aquificae*, *Deinococcus-Thermus*, some genera from *Cyanobacteria* and *Crenarchaeota* were highly dependent on temperature. While, the genus *Halobacterium* from *Euryarchaeota*, unclassified *Thaumarchaeota*, *Actinobacteria* and *Cyanobacteria* have shown significant correlation with salinity and were observed in greater abundances in the two saline hot springs, Garbanabra and Gelti. *Firmicutes* and unclassified *Rhodocylaceae* were significantly higher in the microbial mat samples, while the Candidate Divisions and *Archaea* were prominent in the wet sediment samples.

## Additional files

Additional file 1: Table S1. Physicochemical analysis of the water and wet sediment samples collected from the five hot springs in Eritrea. (DOCX 16 kb)

Additional file 2: Figure S1. Hierarchical clustering of the physiochemical attributes of the hot springs based on Euclidean distance matrix of the water (A) and wet sediment (B) (TIFF 325 kb)

Additional file 3: Table S2. Overview of sequence datasets.s (DOCX 14 kb)

Additional file 4: Figure S2. Rarefaction curves of OTUs from amplicon samples collected from the five hot springs in Eritrea. The number of OTUs is plotted in relation to sub-sampled sequence datasets size (number of reads). The first letters of the sample names refer to the five hot springs (A = Akwar, E = Elegedi, G = Garbanabra, J = Gelti and M = Maiwooi), while the second letters are for sample types (A = Microbial mat, S = wet sediment, and W = water). (TIFF 1023 kb)

Additional file 5: Table S3. Abundance of microbial taxa at phyla level in various samples collected from the five hot springs in Eritrea. The first letters of the sample names refer to the five hot springs (A = Akwar, E = Elegedi, G = Garbanabra, J = Gelti and M = Maiwooi), while the second letters are for sample types (A = microbial mat, S = wet sediment, and W = water). (XLSX 10 kb)

Additional file 6: Table S4. Abundance of bacteria at family level in various samples collected from the five hot springs in Eritrea. The first letters of the sample names refer to the five hot springs (A = Akwar, E = Elegedi, G = Garbanabra, J = Gelti and M = Maiwooi), while the second letters are for sample types (A = microbial mat, S = wet sediment, and W = water). (XLSX 30 kb)

Additional file 7: Table S5. Abundance of archaea at order level in various samples collected from the five hot springs in Eritrea. The first letters of the sample names refer to the five hot springs (A = Akwar, E = Elegedi, G = Garbanabra, J = Gelti and M = Maiwooi), while the second letters are for sample types (A = microbial mat, S = wet sediment, and W = water). (XLS 29 kb)

## Abbreviations

ANOSIM: Analysis of Similarity
ARISA: Automated Ribosomal Space Analysis
BLAST: Basic Local Alignment Search Tool
EC: Electrical Conductivity
GPS: Global Positioning System
HTS: High Throughput Sequencing
NCBI: National Centre for Biotechnology Information
NMDS: Non-metric multidimensional Scaling
ppm: Parts per million
QIIME: Qualitative Insight into Microbial Ecology
RDA: Redundancy Analysis
RDP: Ribosomal Database Project
SSU: Small Subunit
T (°C): Temperature in degree Celsius
TRFLP: Terminal Restriction Fragment Length Polymorphism
US: United States
YNP: Yellowstone National Park

## References

[1] Yohannes E. Geothermal Exploration in Chile: Country Update: Proc. World Geotherm. Congr, Melbourne; 2015. p. 1–11.

[2] Yohannes E. Geothermal exploration in Eritrea. Explor. Geotherm. Resouces, Oct. 29-November 19, 2010, Kenya. 2010.

[3] Abraha M (2005). Geothermal exploration opportunities in Eritrea.

[4] Eloe-Fadrosh EA, Paez-Espino D, Jarett J, Dunfield PF, Hedlund BP, Dekas AE (2016). Global metagenomic survey reveals a new bacterial candidate phylum in geothermal springs. Nat Commun.

[5] Brock TD, Freeze H (1969). *Thermus aquaticus* gen. n. and sp. n., a nonsporulating extreme thermophile. J Bacteriol.

[6] Head IM, Saunders JR, Pickup RW (1998). Microbial evolution, diversity, and ecology: A decade of ribosomal RNA analysis of uncultivated microorganisms. Microb Ecol.

[7] Liu WT, Marsh TL, Cheng H, Forney LJ (1997). Characterization of microbial diversity by determining terminal restriction fragment length polymorphisms of genes encoding 16S rRNA. Appl Environ Microbiol.

[8] Fisher MM, Triplett EW (1999). Automated Apporach for Ribosomal Intergenic Spacer Analysis of microbial diversity and its application to freshwater bacterial communities. Appl Environ Microbiol.

[9] Curtis TP, Head IM, Lunn M, Woodcock S, Schloss PD, Sloan WT (2006). What is the extent of prokaryotic diversity?. Philos Trans R Soc Lond B Biol Sci.

[10] Andersson AF, Lindberg M, Jakobsson H, Bäckhed F, Nyrén P, Engstrand L (2008). Comparative analysis of human gut microbiota by barcoded pyrosequencing. PLoS One.

[11] Hamady M, Walker JJ, Harris JK, Gold NJ, Knight R (2008). Error-correcting barcoded primers allow hundreds of samples to be pyrosequenced in multiplex. Nat Methods.

[12] Herlemann DPR, Labrenz M, Jürgens K, Bertilsson S, Waniek JJ, Andersson AF (2011). Transitions in bacterial communities along the 2000 km salinity gradient of the Baltic Sea. Int Soc Microb Ecol J.

[13] Sogin ML, Morrison HG, Huber JA, Welch DM, Huse SM, Neal PR (2006). Microbial diversity in the deep sea and the underexplored “rare biosphere.”. Proc Natl Acad Sci U S A.

[14] Eiler A, Heinrich F, Bertilsson S (2012). Coherent dynamics and association networks among lake bacterioplankton taxa. Int Soc Microb Ecol J.

[15] Saxena R, Dhakan DB, Mittal P, Waiker P, Chowdhury A, Ghatak A, et al. Metagenomic analysis of hot springs in Central India reveals hydrocarbon degrading thermophiles and pathways essential for survival in extreme environments. Front Microbiol. 2017;7 10.3389/fmicb.2016.02123 PMC5214690 28105025

[16] Mehetre GT, Paranjpe A, Dastager SG, Dharne MS (2016). Investigation of microbial diversity in geothermal hot springs in Unkeshwar, India, based on 16S rRNA amplicon metagenome sequencing. Genome Announc.

[17] Singh A, Subudhi E (2016). Structural insights of microbial community of Deulajhari (India) hot spring using 16s-rRNA based metagenomic sequencing. Genom Data.

[18] Tatio E, Field G, Engel AS, Johnson LR, Porter ML (2013). Arsenite oxidase gene diversity among chloroflexi and proteobacteria from El Tatio geyser field, Chile. FEMS Microbiol Ecol.

[19] Song Z-Q, Wang F-P, Zhi X-Y, Chen J-Q, Zhou E-M, Liang F (2013). Bacterial and archaeal diversities in Yunnan and Tibetan hot springs, China. Environ Microbiol.

[20] Chan CS, Chan KG, Tay YL, Chua YH, Goh KM (2015). Diversity of thermophiles in a Malaysian hot spring determined using 16S rRNA and shotgun metagenome sequencing. Front Microbiol.

[21] Huang Q, Jiang H, Briggs BR, Wang S, Hou W, Li G (2013). Archaeal and bacterial diversity in acidic to circumneutral hot springs in the Philippines. FEMS Microbiol Ecol.

[22] Coman C, Drugǎ B, Hegedus A, Sicora C, Dragoş N (2013). Archaeal and bacterial diversity in two hot spring microbial mats from a geothermal region in Romania. Extremophiles.

[23] Inskeep WP, Jay ZJ, Tringe SG, Herrgard MJ, Rusch DB, Members YMPSC and WG (2013). The YNP metagenome project: Environmental parameters responsible for microbial distribution in the yellowstone geothermal ecosystem. Front Microbiol.

[24] Kambura AK, Mwirichia RK, Kasili RW, Karanja EN, Makonde HM, Boga HI (2016). Bacteria and Archaea diversity within the hot springs of Lake Magadi and Little Magadi in Kenya. BMC Microbiol.

[25] Sambrok J, Russell D, Argentine J, Irwin N, Janssen KA, Curtis S, Zierler M, Mclnerny N (2001). Molecular cloning: A laboratory manual.

[26] Caporaso JG, Lauber CL, Walters WA, Berg-Lyons D, Huntley J, Fierer N (2012). Ultra-high-throughput microbial community analysis on the Illumina HiSeq and MiSeq platforms. Int Soc Microb Ecol J.

[27] Yu K, Zhang T (2012). Metagenomic and metatranscriptomic analysis of microbial community structure and gene expression of activated sludge. PLoS One.

[28] Reeder J, Knight R (2010). Rapid denoising of pyrosequencing amplicon data: exploiting the rank-abundance distribution. Nat Methods.

[29] Gontcharova V, Youn E, Sun Y, Wolcott RD, Dowd SE (2010). A comparison of bacterial composition in diabetic ulcers and contralateral intact skin. Open Microbiol J.

[30] DeSantis TZ, Hugenholtz P, Larsen N, Rojas M, Brodie EL, Keller K (2006). Greengenes, a chimera-checked 16S rRNA gene database and workbench compatible with ARB. Appl Environ Microbiol.

[31] R Development Core Team (2012). R: A language and environment for statistical computing.

[32] Caporaso JG, Kuczynski J, Stombaugh J, Bittinger K, Bushman FD, Costello EK (2010). QIIME allows analysis of high- throughput community sequencing data. Nat Methods.

[33] Oksanen AJ, Blanchet FG, Friendly M, Kindt R, Legendre P, Mcglinn D (2016). Vegan: Community Ecology Package. R package version 2.3–5.

[34] Huang Q, Dong CZ, Dong RM, Jiang H, Wang S, Wang G (2011). Archaeal and bacterial diversity in hot springs on the Tibetan Plateau, China. Extremophiles.

[35] Tekere M, Lötter A, Olivier J, Jonker N, Venter S (2011). Metagenomic analysis of bacterial diversity of Siloam hot water spring, Limpopo, South Africa. Afr J Biotechnol.

[36] De León KB, Gerlach R, Peyton BM, Fields MW (2013). Archaeal and bacterial communities in three alkaline hot springs in Heart Lake Geyser Basin, Yellowstone National Park. Front Microbiol.

[37] Sharma A, Paul D, Dhotre D, Jani K, Pandey A, Shouche YS (2017). Deep sequencing analysis of bacterial community structure of Soldhar hot spring, India. Microbiology.

[38] Ghelani A, Patel R, Mangrola A, Dudhagara P (2015). Cultivation-independent comprehensive survey of bacterial diversity in Tulsi Shyam hot springs, India. Genom Data.

[39] Cheng L, Qiu T, Yin X, Wu X, Hu G, Deng Y (2007). *Methermicoccus shengliensis* gen. nov., sp. nov., a thermophilic, methylotrophic methanogen isolated from oil-production water, and proposal of Methermicoccaceae fam. nov.. Int J Sytematic Evol Microbiol.

[40] Inskeep WP, Rusch DB, Jay ZJ, Herrgard MJ, Kozubal MA, Richardson TH (2010). Metagenomes from high-temperature chemotrophic systems reveal geochemical controls on microbial community structure and function. PLoS One.

[41] Hou W, Wang S, Dong H, Jiang H, Briggs BR, Peacock JP (2013). A comprehensive census of microbial diversity in hot springs of tengchong, Yunnan Province China using 16S rRNA gene pyrosequencing. PLoS One.

[42] Tobler DJ, Benning LG (2011). Bacterial diversity in five Icelandic geothermal waters: temperature and sinter growth rate effects. Extremophiles.

[43] Blank CE, Cady SL, Pace NR (2002). Microbial composition of near-boiling silica-depositing thermal springs throughout Yellowstone National Park. Appl Environ Microbiol.

[44] Nakagawa T, Fukui M (2003). Molecular characterization of community structures and sulphur metabolism within microbial streamers in Japanese hot springs. Appl Environ Microbiol.

[45] Gumerov VM, Mardanov AV, Beletsky AV, Bonch-Osmolovskaya EA, Ravin NV (2011). Molecular analysis of microbial diversity in the Zavarzin Spring, Uzon Caldera, Kamchatka. Microbiology.

[46] Vick TJ, Dodsworth JA, Costa KC, Shock EL, Hedlund BP (2010). Microbiology and geochemistry of Little Hot Creek, a hot spring environment in the Long Valley Caldera. Geobiology.

[47] Costa KC, Nabarro JB, Shock EL, Zhang CL, Soukup D, Hedlund BP (2009). Microbiology and geochemistry of great boiling and mud hot springs in the United States Great Basin. Extremophiles.

[48] Purcell D, Sompong U, Yim LC, Barraclough TG, Peerapornpisal Y, Pointing SB (2007). The effects of temperature, pH and sulphide on the community structure of hyperthermophilic streamers in hot springs of northern Thailand. FEMS Microbiol Ecol.

[49] Wang S, Hou W, Dong H, Jiang H, Huang L, Wu G (2013). Control of temperature on microbial community structure in hot springs of the Tibetan Plateau. PLoS One.

[50] Nobre MF, Truper HG, Da Costa MS (1996). Transfer of Thermus ruber (Longinova et al. 1984), Thermus silvanus (Tenriro et al. 1995), and Thermus chliarophilus (Tenreiro et al.1995) to Meiothermus gen. nov. as Meiothermus ruber comb. nov., Meiothermus silvanuscom nov., and Meiothermus chliarophilus comb. nov., respectively, and emendation of the genus Thermus. Int J Syst Evol Microbiol.

[51] Thomsen JK, Cox RP. Upper temperature limits for growth and diazotrophy in the thermophilic cyanobacterium HTF *Chlorogloeopsis*. Arch Microbiol. 1993;159:423–7.

[52] Huber H, Stetter KO, Garrity GM, Boone DR, Castenholz RW (2001). Bergey’s manual of systematic bacteriology. The archea and deeply branching and phototrophic bacteria.

[53] Straub KL, Schönhuber WA, Buchholz-Cleven BEE, Schink B (2004). Diversity of ferrous iron-oxidizing, nitrate-reducing bacteria and their involvement in oxygen-independent iron cycling. Geomicrobiol J.

[54] Busse H, Ka P (2002). *Thermomonas haemolytica* gen. nov., sp. nov., a γ-proteobacterium from kaolin slurry. Int J Syst Evol Microbiol.

[55] Lebedeva EV, Alawi M, Fiencke C, Namsaraev B, Bock E, Spieck E (2005). Moderately thermophilic nitrifying bacteria from a hot spring of the Baikal rift zone. FEMS Microbiol Ecol.

[56] Golvacheva RS (1976). Thermophilic nitrifying bacteria from hot springs. Mikrobiologiia.

[57] Doughari HJ, Ndakidemi PA, Human IS, Benade S (2011). The ecology, biology and pathogenesis of *Acinetobacter spp*.: an overview. Microbes Environ.

[58] Fang L, Chen L, Liu Y, Tao W, Zhang Z, Liu H (2015). Planktonic and sedimentary bacterial diversity of Lake Sayram in summer. Microbiology.

