# Supplementary material for: Evaluation of prokaryotic diversity of five hot springs in Eritrea
Source: BMC Microbiol. 2017 Sep 22;17:203. doi: 10.1186/s12866-017-1113-4 (PMC5610464; doi:10.1186/s12866-017-1113-4)
Supplement: Supplementary file 1 — Physicochemical analysis of the water and wet sediment samples collected from the five hot springs in Eritrea. (DOCX 16 kb) [file 12866_2017_1113_MOESM1_ESM.docx]

| **Parameter** | **Akwar** | | **Elegedi** | | **Garbanabra** | | **Gelti** | | **Maiwooi** | |
| --- | --- | --- | --- | --- | --- | --- | --- | --- | --- | --- |
|  | **water** | **sediment** | **water** | **sediment** | **water** | **sediment** | **water** | **sediment** | **water** | **sediment** |
| EC (mS/cm) | 9290±116 | ND | 2230±112 | ND | 24400±696 | ND | 15717±148 | ND | 1317±117 | ND |
| TDS (mg/L) | 628±26 | ND | 1546±58 | ND | 15552±585 | ND | 9290±336 | ND | 543±38 | ND |
| T (°C) | 49.5±0.9 | ND | 100±0.0 | ND | 51.3±0.6 | ND | 52.6±1.4 | ND | 51.4±0.5 | ND |
| pH | 7.22±0.01 | ND | 7.01±0.02 | ND | 7.05±0.01 | ND | 7.19±0.01 | ND | 7.54±0.02 | ND |
| SiO2 (mg/L) | 100.3±1.5 | ND | 97.4±2.9 | ND | 121.7±8.8 | ND | 116.2±3.5 | ND | 78.1±3.2 | ND |
| Na^+^ (mg/L) | 188±7 | 251±13 | 11±1 | 62.2±1.4 | 3800±101 | 1160± | 2378±89 | 862±36 | 227±10 | 111±6 |
| K^+^ (mg/L) | 104±3 | 134±6 | 12±2 | 57±5 | 198±11 | 473±23 | 119±3 | 368±6 | 13±2 | 39±3 |
| Ca^+2^ (mg/L | 19±2 | 219±5 | 157±8 | 340±7 | 1653±87 | 1430±76 | 943±13 | 368±11 | 52±4 | 54±6 |
| Mg^+2^ (mg/L) | 0.5±0.2 | 21.2±0.8 | 39.0±3.1 | 21.3±2.9 | 151.0±7.1 | 152±9.9 | 89.0±3.1 | 124±4.4 | 5.6±1.1 | 35.1±3.5 |
| NH**_4_**^+^ (mg/L) | 0.11±0.01 | ND | 196.31±2.7 | ND | 0.03±0.00 | ND | 0.06±0.01 | ND | 0.03±0.00 | ND |
| NO**_3_**^-^ (mg/L) | 1.50±0.26 | ND | 0.01±0.00 | ND | 54.83±7.73 | ND | 56.33±8.01 | ND | 0.19±0.02 | ND |
| P(O) (mg/L | ND | 0.37±0.06 | ND | 4.26±0.79 | ND | 2.55±0.87 | ND | 1.81±0.22 | ND | 0.72±0.10 |
| Fe^+2^ (mg/L) | <0.01 | 38.0±2.1 | 0.8±0.0 | 404.0±8.8 | <0.01 | 153±12.1 | <0.01 | 124±11.3 | <0.01 | 39.6±3.3 |
| Mn (mg/ L) | <0.01 | 17.5±1.4 | 3.0±0.45 | 101.0±6.7 | 20.1±1.4 | 57.0±3.5 | <0.01 | 245.0±8.3 | <0.01 | 35.6±4.2 |
| Zn (mg/L) | ND | 6.9±1.1 | ND | 44.4±3,2 | ND | 5.9±0.8 | ND | 5.1±0.6 | ND | 1.7±0.0 |
| Cu (mg/L) | ND | 1.95±0.01 | ND | 5.28±0.21 | ND | 1.39±0.02 | ND | 1.28±0.01 | ND | 0.75±0.00 |
| Cl^-^ (mg/ L) | 17±1 | ND | 1±0 | ND | 5945±121 | ND | 5134±116 | ND | 71±6 | ND |
| SO**_4_**^-2^ (mg/L) | 42.4±3.5 | ND | 949.7±75.5 | ND | 272.7±12.7 | ND | 220.3±7.5 | ND | 75.3±1.2 | ND |
| S (mg/L) | ND | 658±18 | ND | 728±22 | ND | 61±2 | ND | 92±5 | ND | 15±1 |
| CO**_3_**^-2^ (mg/L) | 22.3±0.3 | ND | 21.3±0.3 | ND | 28.7±1.5 | ND | 23.0±0.2 | ND | 19.5±0.4 | ND |
| HCO**_3_** (mg/L) | 341.0±9.5 | ND | 174.7±6.3 | ND | 27.7±0.7 | ND | 29.3±1.8 | ND | 394.3±9.8 | ND |
| F^-^ (mg/L) | 8.20±1.30 | ND | 1.18±0.20 | ND | 2.87±0.40 | ND | 2.87±0.70 | ND | 6.48±1.10 | ND |
| B^-^ (mg/L) | 0.33±0.02 | 11.50±1.11 | 0.02±0.00 | 0.20±0.01 | 2.21±0.06 | 1.60±0.08 | 1.55±0.04 | 1.00±0.00 | 0.25±0.00 | 10.20±0.93 |
| Br^-^ (mg/L) | 0.90±0.01 | ND | <0.001 | ND | 74.80±6.76 | ND | 45.20±2.21 | ND | 0.38±0.04 | ND |
