# Supplementary figures and images for: Evaluation of prokaryotic diversity of five hot springs in Eritrea
Source: BMC Microbiol. 2017 Sep 22;17:203. doi: 10.1186/s12866-017-1113-4 (PMC5610464; doi:10.1186/s12866-017-1113-4)

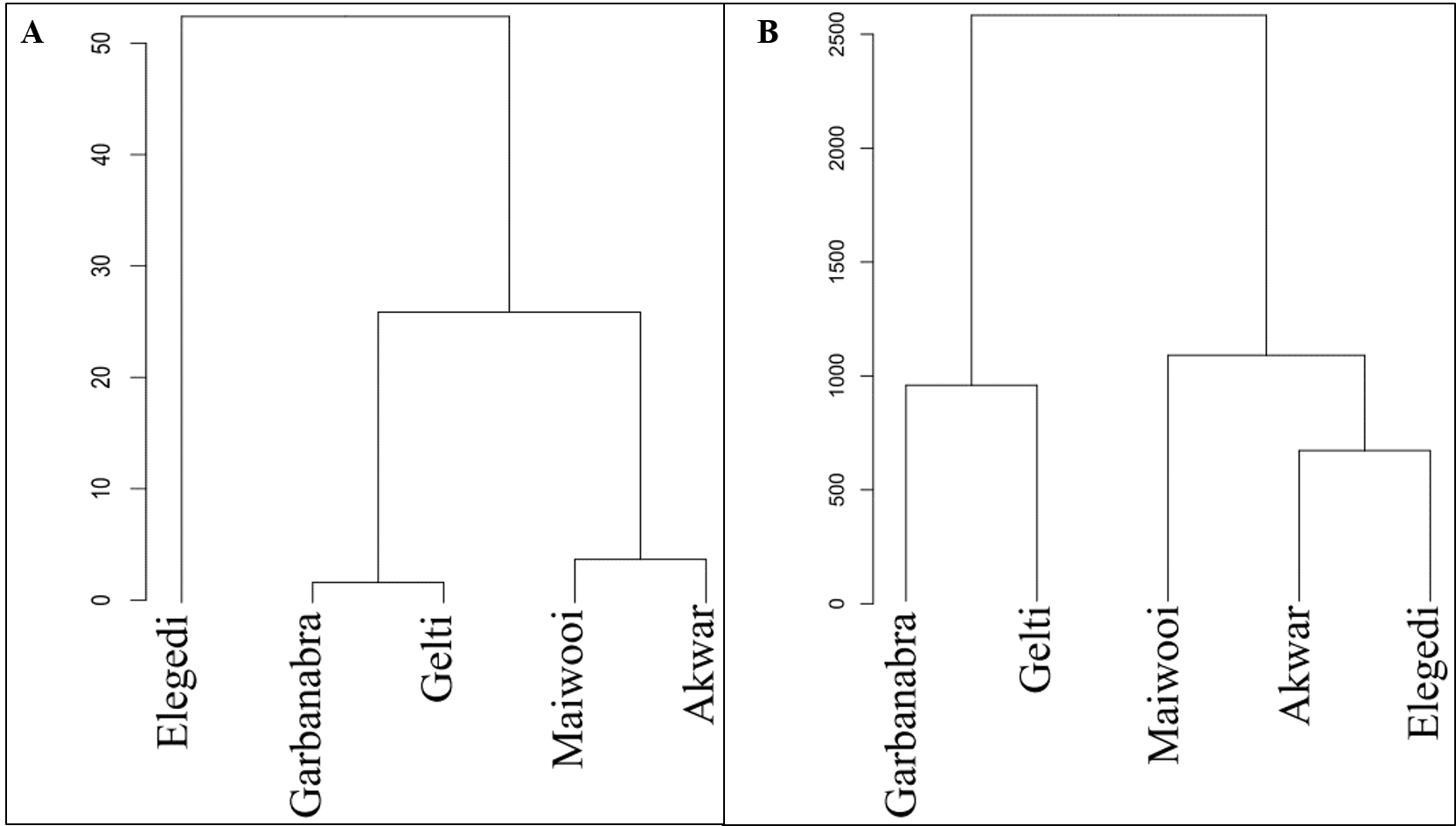

Supplement: Supplementary file 2 — Hierarchical clustering of the physiochemical attributes of the hot springs based on Euclidean distance matrix of the water (A) and wet sediment (B) (TIFF 325 kb) [file 12866_2017_1113_MOESM2_ESM.tif]

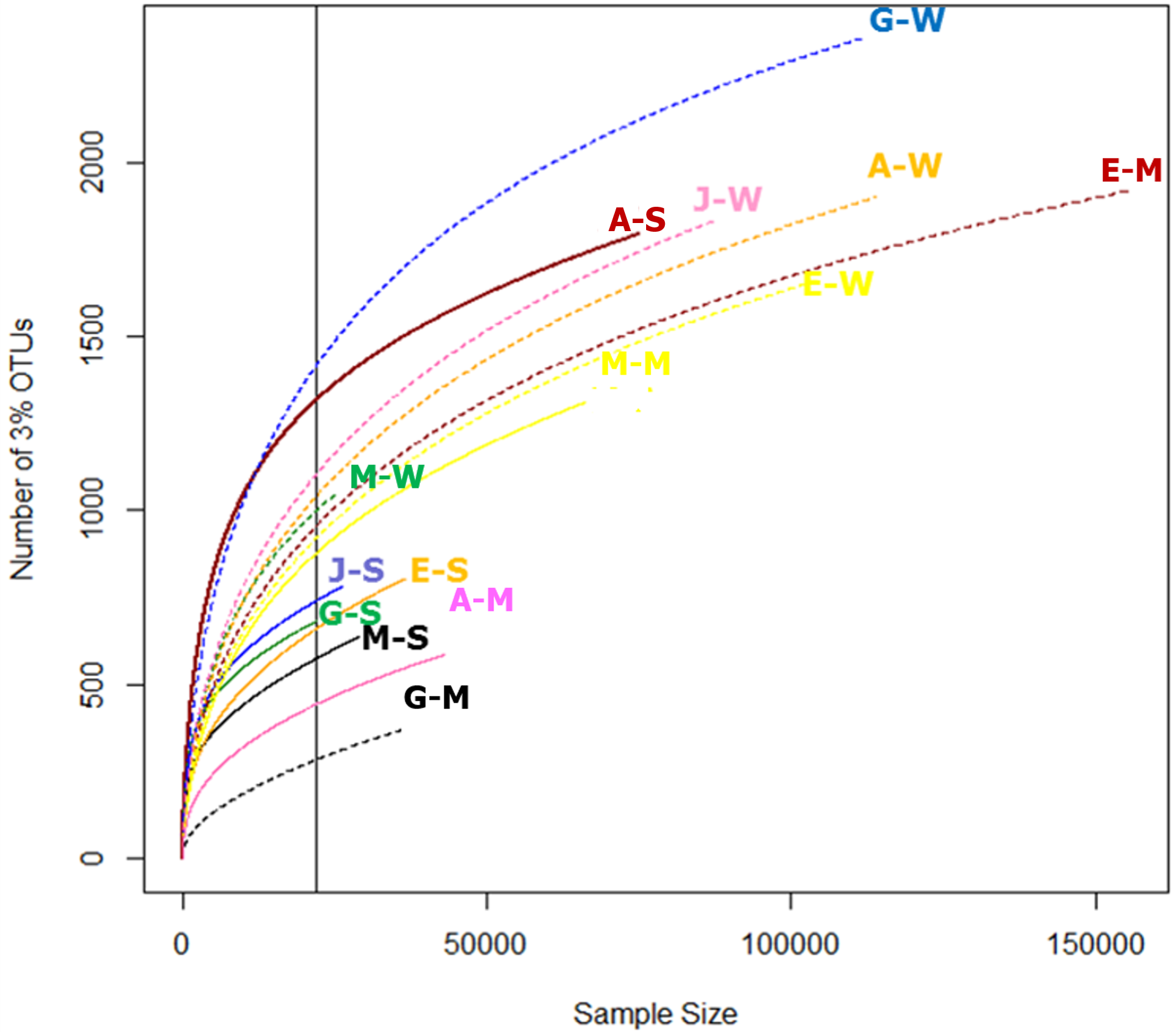

Supplement: Supplementary file 4 — Rarefaction curves of OTUs from amplicon samples collected from the five hot springs in Eritrea. The number of OTUs is plotted in relation to sub-sampled sequence datasets size (number of reads). The first letters of the sample names refer to the five hot springs (A = Akwar, E = Elegedi, G = Garbanabra, J = Gelti and M = Maiwooi), while the second letters are for sample types (A = Microbial mat, S = wet sediment, and W = water). (TIFF 1023 kb) [file 12866_2017_1113_MOESM4_ESM.tif]
