# Supplementary material for: Evaluation of prokaryotic diversity of five hot springs in Eritrea
Source: BMC Microbiol. 2017 Sep 22;17:203. doi: 10.1186/s12866-017-1113-4 (PMC5610464; doi:10.1186/s12866-017-1113-4)
Supplement: Supplementary file 3 — Overview of sequence datasets.s (DOCX 14 kb) [file 12866_2017_1113_MOESM3_ESM.docx]

| Hot spring | Sample Type | Raw reads | Raw reads  >200 bp | Chimera reads (%) | Number of OTUs | Abundance | Shannon index | Inverse Simpson index |
| --- | --- | --- | --- | --- | --- | --- | --- | --- |
| Akwar | Mats | 46883 | 46877 | 8.19 | 587 | 43036 | 3.70 | 15.74 |
|  | Water | 134969 | 134926 | 15.70 | 1898 | 113743 | 4.70 | 34.72 |
|  | Wet sediment | 86401 | 86366 | 13.07 | 1794 | 75075 | 5.62 | 76.00 |
| Elegedi | Mats | 183047 | 183005 | 14.87 | 1919 | 155789 | 4.59 | 33.48 |
|  | Water | 122558 | 122529 | 14.85 | 1662 | 104328 | 4.52 | 30.62 |
|  | Wet sediment | 43433 | 43424 | 16.03 | 805 | 36465 | 4.24 | 18.69 |
| Garbanabra | Mats | 40479 | 40469 | 8.82 | 376 | 36900 | 2.68 | 7.91 |
|  | Water | 148471 | 148385 | 24.89 | 2354 | 111458 | 5.52 | 94.28 |
|  | Wet sediment | 25158 | 25150 | 12.16 | 684 | 22091 | 5.34 | 77.38 |
| Gelti | Water | 102909 | 102865 | 14.90 | 1834 | 87537 | 5.21 | 77.52 |
|  | Wet sediment | 29916 | 29907 | 11.76 | 784 | 26390 | 5.11 | 70.35 |
| Maiwooi | Mats | 76598 | 76577 | 13.60 | 1311 | 66165 | 4.17 | 22.33 |
|  | Water | 29435 | 29419 | 13.03 | 1054 | 25586 | 4.76 | 32.63 |
|  | Wet sediment | 32441 | 32433 | 10.18 | 639 | 29132 | 4.81 | 44.59 |
| **Total** | | **1,102,698** | **1,102,332** | **-** | - | **933,695** | - | - |
